# Quantitative Real-Time PCR Assay for the Detection of *Pectobacterium parmentieri*, a Causal Agent of Potato Soft Rot

**DOI:** 10.3390/plants10091880

**Published:** 2021-09-10

**Authors:** Anna A. Lukianova, Peter V. Evseev, Alexander A. Stakheev, Irina B. Kotova, Sergey K. Zavriev, Alexander N. Ignatov, Konstantin A. Miroshnikov

**Affiliations:** 1 Shemyakin-Ovchinnikov Institute of Bioorganic Chemistry, Russian Academy of Sciences, Miklukho-Maklaya Str., 16/10, 117997 Moscow, Russia; a.al.lukianova@gmail.com (A.A.L.); petevseev@gmail.com (P.V.E.); stakheev.aa@gmail.com (A.A.S.); szavriev@ibch.ru (S.K.Z.); 2 Department of Biology, Lomonosov Moscow State University, Leninskie Gory, 1, Bldg. 12, 119234 Moscow, Russia; kira1959@gmail.com; 3 AgroTechnical Institute, RUDN University, Miklukho-Maklaya Str., 6, 117198 Moscow, Russia; an.ignatov@gmail.com

**Keywords:** *Pectobacterium parmentieri*, qPCR, bacterial taxonomy, bacterial identification, sensitivity, soft rot, pathogen detection

## Abstract

*Pectobacterium parmentieri* is a plant-pathogenic bacterium, recently attributed as a separate species, which infects potatoes, causing soft rot in tubers. The distribution of *P. parmentieri* seems to be global, although the bacterium tends to be accommodated to moderate climates. Fast and accurate detection systems for this pathogen are needed to study its biology and to identify latent infection in potatoes and other plant hosts. The current paper reports on the development of a specific and sensitive detection protocol based on a real-time PCR with a TaqMan probe for *P. parmentieri*, and its evaluation. In sensitivity assays, the detection threshold of this protocol was 10^2^ cfu/mL on pure bacterial cultures and 10^2^–10^3^ cfu/mL on plant material. The specificity of the protocol was evaluated against *P. parmentieri* and more than 100 strains of potato-associated species of *Pectobacterium* and *Dickeya*. No cross-reaction with the non-target bacterial species, or loss of sensitivity, was observed. This specific and sensitive diagnostic tool may reveal a wider distribution and host range for *P. parmentieri* and will expand knowledge of the life cycle and environmental preferences of this pathogen.

## 1. Introduction

The potato (*Solanum tuberosum*) is one of the most important crops in the world. The world market for potato production exceeds 388 million tons per year (https://www.potatopro.com/world/potato-statistics (accessed on 7 April 2021)) and per capita consumption in Russia exceeds 110 kg (https://www.potatopro.com/russian-federation/potato-statistics (accessed on 7 April 2021)). Therefore, research related to optimising potato production, increasing yields and reducing losses associated with plant diseases and other factors is essential and urgent. Among the challenges faced by potato growers is potatoes’ spoilage as a result of bacterial infections. In particular, the development of rot on tubers during storage and transportation can lead to severe losses—up to half of the harvest [1]. The leading cause of blackleg and soft rot in potatoes is the bacteria of the Pectobacteriaceae family, namely the group of Soft Rot Pectobacteriaceae (SRP), comprising phytopathogens of the genera *Pectobacterium* and *Dickeya* [2]. One of the representatives of this group is *P. parmentieri*.

*P. parmentieri* (Ppa) was first described by Khayi et al. in 2016. It is a species closely related to the previously known pathogen of Japanese horseradish, *P. wasabiae* (Pwa). Several Pwa strains, isolated from potatoes and which cause soft rot, have been scrutinised and finally reclassified as new species [3]. Later on, Ppa was identified among potato pathogens in circulation in Europe and Russia [4,5], Africa [6], Asia [7,8] and America [9,10]. Many strains isolated from potatoes earlier, and initially attributed as Pwa or *Pectobacterium carotovorum* subsp. *Carotovorum*, were proved to represent Ppa.

Thus, *P. parmentieri* can be considered as a worldwide pathogen (https://www.cabi.org/isc/datasheet/48069201 (accessed on 17 May 2021). Strains of Ppa studied are rather diverse [11,12], and two other species related to Ppa/Pwa, *P. polonicum* [13] and *P. punjabense* [14] have been established recently. A recent study of the distribution of *P. punjabense* in Europe [15] demonstrated the need for an appropriate method for discriminatory quantitative diagnostics for newly established SRP species.

Many PCR-based methods have already been developed for generalised and species-specific detection of SRP (reviewed in [16,17]). The accumulation of data on bacterial genomics and taxonomic redistributions has encouraged the design of an updated method for the specific diagnosis of newly established SRP species, particularly Ppa. Earlier, PCR diagnostic methods were proposed for Pwa detection, based on the amplification of the phytase/phosphatase (appA) gene [18] or tyrosine-aspartate (YD) repeat region [19]. Both of these assays enabled scientists to discriminate Ppa/Pwa from *P. carotovorum* and other SRP, but not between the former species. The analysis currently used in phytodiagnostics enables an assumption of the approximate specificity of the pathogen, taking into account the source of the isolation of the strain [4]. However, it still does not allow for species-specific detection and is somewhat outdated, due to the changed understanding of the taxonomy of the group. Recently, the authors developed a pipeline for searching unique sequences for genomic groups and tested it in the context of *P. atrosepticum*, a genetically distinct species of SRP [20]. This paper describes how this workflow can be used to design a quantitative real-time PCR assay to discriminate closely related species. The aim of the study was the development of a species-specific detection system for *P. parmentieri.*

## 2. Results

### 2.1. Phylogenetic Analysis

By early 2021, more than 200 bacterial genomes deposited in NCBI GenBank represented the family *Pectobacteriaceae*. *P. parmentieri* was represented by 30 complete and high coverage draft genomes (strains CFIA102, IFB5408, IFB5427, IFB5432, IFB5441, IFB5485, IFB5486, IFB5597, IFB5604, IFB5605, IFB5619, IFB5623, IFB5626, IPO1955, NY1532B, NY1533B, NY1540A, NY1548A, NY1584A, NY1585A, NY1587A, NY1588A, NY1712A, NY1722A, PB20, QK5, RNS-08-42-1A (type strain), SS90, WC19161 and WPP163).

**Figure 1:**
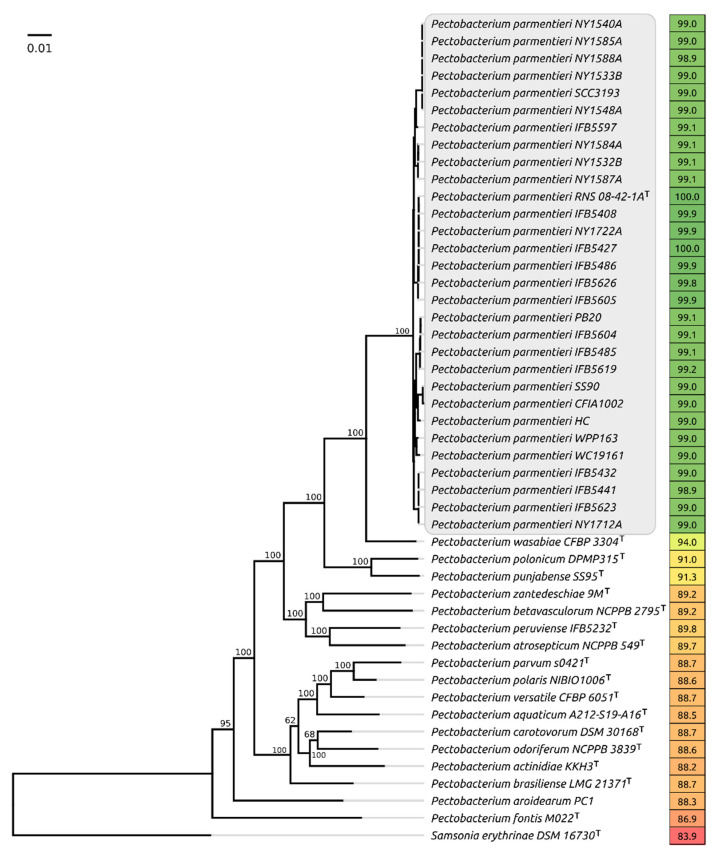
Phylogenetic tree based on the concatenated nucleotide sequences of 92 conservative genes, including the genes of ribosomal proteins and the proteins essential for the transcription and translation processes. Bootstrap support values are shown above their branch as a percentage of 1000 replicates. The scale bar shows 0.01 estimated substitutions per site, and the tree was rooted to *Samsonia erythrinae DSM 16730*. Average nucleotide identity (ANI) values compared to *P. parmentieri* RNA 08-42-1A type strain are shown to the right of the organism name and coloured according to a heat map scale, where a green colour corresponds to the highest value and a red colour corresponds to the lowest value.

Whole-genome comparisons made with orthoANI (Figure 1) demonstrate that all the strains assigned to *P. parmentieri* possess a close genome similarity, demonstrating high overall average nucleotide similarity (ANI) of 98.9% and above when compared to the type species, whereas the comparable ANI values of non-*parmentieri Pectobacterium* species lies in the range of 87–94%. The concatenated core genes phylogeny also places Ppa strains in a distinct clade (Figure 1).

### 2.2. Search for Species-Specific Primers

The search for species-specific sequences was carried out using the workflow described in a previous study [20]. Briefly, this workflow splits the genome of the type Ppa strain into short sections, then each section is compared with a negative database of “non-target” genomes and a positive database of “target genomes” and, as a result, regions are identified that occur in all Ppa genomes and are not found in genomes of other species.

Using this search, a set of unique Ppa species-specific sites was obtained. Regions belonging to the areas of the genome encoding no genes were manually rejected. Next, several potentially suitable sites within the housekeeping genes were selected for further preliminary testing in the conventional PCR mode (Section 2.3) and a further selection of the most appropriate sequence for qPCR analysis development was made (Section 2.4). Primers and probes were designed for these sites. Table 1 shows the sequences of primers, probe and amplicon for detection based on the ankyrin repeat domain-containing protein sequence that showed the best results and was therefore selected for further study.

**Figure 2:**
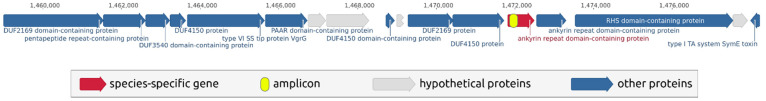
Region in the *P. parmentieri* RNS 08-42-1AT genome containing a species-specific sequence. The scheme was visualised using Geneious Prime 2021.2.2 (https://www.geneious.com, accessed on 20 January 2021).

The selected species-specific sequence belongs to an ankyrin repeat domain-containing protein that is located adjacent to the components of a type VI secretion system. Interestingly, an avirulence factor was located several genes upstream of the locus shown in Figure 2. A type VI secretion system is important for plant-associated bacteria, including the *Pectobacterium* species. It contributes to virulence and grants fitness and colonisation advantages *in planta* [21]. It might be suggested that the gene containing the species-specific sequence is important for the bacterium. The sequence search conducted with BLAST using an nr/nt database confirmed that the chosen amplicon did not have close homologues in other organisms.

### 2.3. Primary Analysis by Conventional PCR

For the initial assessment of the applicability of the primers obtained for the purpose of species-specific PCR detection, a conventional PCR test was carried out on a limited set of strains. The strains marked F… are a part of the local collection of bacterial pathogens associated with potato soft rot. The collection includes comprehensively described type strains, strains with appropriate genomic characterisation and loosely characterised local isolates. The information on the strains used is provided in Supplementary Table S1. The primary testing strain set included several representatives of different Pectobacteriaceae species belonging to the genus *Pectobacterium* (F002, F004, F012, F016, F028, F041, F043, F048, F061, F109, F126, F131, F135, F152, F157, F160, F162, F164, F171, F182, F258), *Dickeya* (F012, F077, F082, F085, F097, F101, F102, F117, F155, F261) and an unrelated pectolytic isolate (F105). In the experiment described in this paper, amplification was expected only for Ppa (F034, F035, F127, F148, F149, F174), and with none of the others.

**Figure 3:**
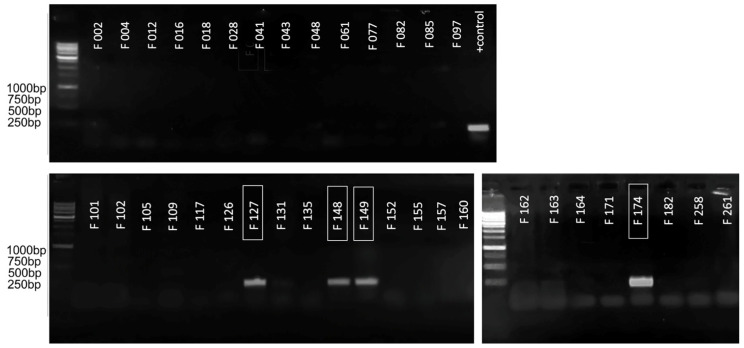
The results of conventional PCR visualised in 1.5% agarose gel. The numbers of the strains belonging to Ppa are marked with a frame. The remaining strains belonging to other species acted as negative controls. The lane designated as “+ control” contained PCR results with test plasmid, which served as a positive control. Evrogen 1 kb Ladder used for the evaluation of amplicons sizes.

Figure 3 shows the results of such an analysis for the amplification of ankyrin repeat domain-containing protein, as a result of which significant amplification was demonstrated only with the target strains (marked in the boxes) and in the absence of false-positive results with all other strains. This enabled the assumption of this site’s suitability for amplification in qPCR mode, and made it possible to proceed to the validation using an extended range of strains.

### 2.4. qPCR Analysis on an Extended Set of Strains

This study involved seven strains previously attributed to being Ppa or Pwa on the basis of genomic sequencing or 16S rRNA gene sequencing. Two more strains were previously identified as Pwa using the diagnostic primer set PhF 5′-GGTTCAGTGCGTCAGGAGAG and PhR 5′-GCGGAGAGGAAGCGGTGAAG [18], which does not distinguish between Pwa and closely related Ppa (№ 1–9, Supplementary Table S1). A test was also conducted for 67 (№ 10–77) isolates of other *Pectobacteriaceae* species and 32 strains (№ 78–109) related to other species associated with crop rot. These strains were isolated from potato rots and passed through McConkey’s medium to exclude *Salmonella* and Gram-positive isolates and SVP medium to ensure the presence of pectolytic activity.

As shown in Supplementary Table S1, all Ppa strains demonstrated a positive PCR signal. Among the strains with alternative Ppa/Pwa attribution (F035 and F178), F035 showed amplification and therefore can be more accurately classified as Ppa, while F178, revealing no positive signal, may be categorised as Pwa.

The historical strain Pwa F007 used in the study did not show any false positive amplification. No positive results were shown for other isolates with pectolytic activity, both Pectobacteriaceae and unrelated ones.

Additionally, in silico analysis using an nt-database did not presume any amplification of plant genomic DNA using the designed primers. No amplification was observed in the PCR reaction in vitro using potato DNA as a template. Thus, the authors are confident that the possibility of cross-amplification with potato DNA was excluded.

### 2.5. Sensitivity

**Figure 4:**
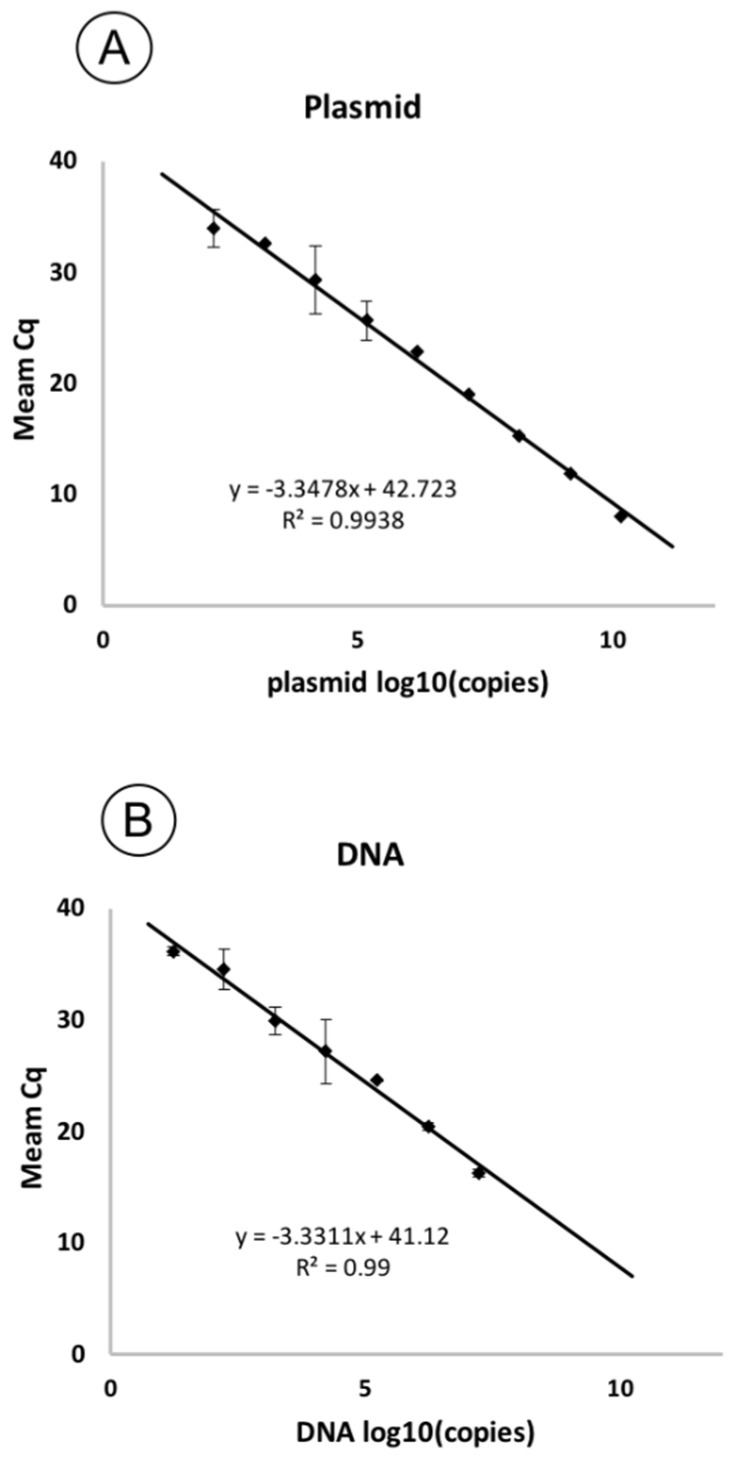
Standard curves showing the dependence of Cq on the concentration of pathogen DNA in the reaction. The curves are plotted based on the threshold cycles obtained for a series of ten-fold dilutions of the plasmid (**A**) and genomic DNA of the F149 strain (**B**). The standard deviation is shown as error bars.

Serially diluted plasmid and genomic DNA were used in qPCR reactions for a sensitivity test. Based on the threshold cycles (Cq) obtained for each concentration of copies in the sample (Table 2), standard curves were plotted. The resulting curves were linear (Figure 4). The correlation coefficient (R^2^) was 0.99 for both curves, with a slope of −3.34 and −3.33 for plasmid and genomic DNA, respectively, corresponding to a PCR efficiency of 98.9% and 99.62%.

**Figure 5:**
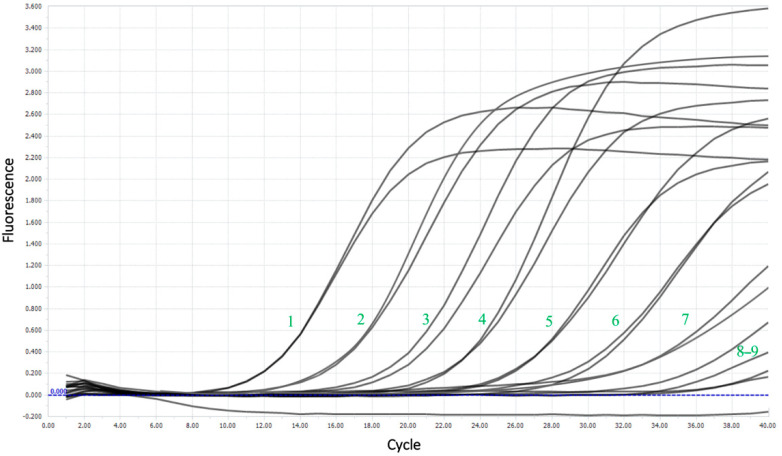
Amplification curves for a sensitivity test using the example of a series of dilutions of plasmid DNA. The numbers represent the dilution number.

The limit of detection (LoD) was nearly 16 copies per reaction, corresponding to 4 × 10^2^ copies/mL. Figure 5 shows the amplification curves for the sensitivity test and the good flare-up of the probe during the reaction, even at high dilutions.

### 2.6. Assays of Plant Samples

To conduct an experiment simulating a pathogen’s detection in infected plants, the tubers of the “Gala” variety were used, one of the most widespread varieties in Russia, and one which is moderately resistant to bacterial diseases. The potatoes were soaked in a 10^6^ cfu/mL suspension of the pathogen for infection and then incubated at 28 °C until the development of soft rot symptoms. On days 3, 4 and 5, a ~100 mg piece of peel was taken from the tubers and total DNA was isolated. Then, qPCR was performed from the DNA obtained, in the same way as in the previous experiments. Control tubers were soaked in a sterile LB medium.

As shown in Table 3, the pathogen was successfully detected in all cases, confirming the possibility of using the analysis to assess the contaminated material. With an increase in the duration of incubation, the titre of bacteria increased proportionally. Amplification was also recorded for the control tuber, indicating a trace presence of the pathogen, which did not lead to noticeable symptoms of rotting.

## 3. Discussion

According to the species definition, Ppa differs from Pwa by its ability to produce acid from melibiose, raffinose, lactose and D-galactose [3]. This feature was used to differentiate Ppa strains isolated from potato in Southern Europe [4]. However, the biochemical tests made the precise diagnostics more laborious and, thus, raised questions about the value of such fine analysis. Besides the obvious purpose of monitoring the causal agents of plant diseases, in order to develop adapted prevention actions in particular countries, regions or climate areas, some fundamental arguments exist.

Information on the role of Ppa in the bacterial pathogenesis of potatoes worldwide is contradictory [22]. According to national monitoring surveys, Ppa occurrence ranges from single, moderate cases [6] to severe breakouts [10]. While wet weather throughout the year is preferred for the development of the pathogen (https://www.cabi.org/isc/datasheet/48069201 (accessed on 17 May 2021)), a broad range of conditions is tolerated. The aggressiveness of Ppa is also debatable. As for other SRP, their pathogenesis relies on the production and secretion of plant cell wall-degrading enzymes, which cause the typical symptoms of soft rot. Enzyme synthesis depends on suitable environmental conditions [23]. Generally, the virulence of Ppa is considered to be moderate. However, a number of studies [24,25] have demonstrated that some strains of *P. parmentieri* can cause fast and severe maceration of tubers and plants comparable with *P. atrosepticum* and *P. brasiliense*, which are considered to be the most aggressive among *Pectobacterium*. It is worth noting that the bacterial community in rotting potato tissues is very complex [26] and may include several different pathogenic species. SRP pathogens may interact antagonistically [27] or synergistically [28] with respect to one another. Therefore, the study of the impact of a particular pathogen on the development of the disease requires quantitative differential identification of the SRP species, particularly with Ppa.

Currently, no effective control agents have been developed to prevent or to treat SRP infections [29,30]. A promising approach is the use of bacteriophages (phages), which are bacterial viruses that infect pathogenic bacteria. A number of successful applications of phage control of plant pathogens, including SRP, have been reported (reviewed in [31,32]). Some phages infecting Ppa have been isolated and investigated [33,34]. An important feature of phage therapy is to have a very selective host range of bacteriophages, usually limited to a bacterial species or even a group of strains within a species. This may be considered to be an advantage, because phage treatment does not affect commensal and endosymbiotic microflora of the plant attacking pathogenic bacteria only. However, scientifically sound use of therapeutic bacteriophages requires fine and precise diagnostics of the causative agent of the disease. Existing assays are often too general for efficient phage application, and more focused methods of discriminating SRP are needed.

Besides pectolytic enzymes, a number of other proteinaceous and carbohydrate factors and signal pathways have been found to participate in bacterial adhesion, the colonising of plant tissue and enhancement of the disease (reviewed in [23]). Essential intracellular effectors have been secreted into the plant cell via secretion systems type III (T3SS), type IV (T4SS) and type VI (T6SS) [35]. An important feature of Ppa/Pwa is the absence of a number of essential genes encoding T3SS in the genome [36,37]. This absence may explain the limited host range of *P. parmentieri*. In such conditions, the role of T6SS and other secretion systems becomes more important [38]. The genomic sequence unique to Ppa that was identified was located adjacent to the T6SS apparatus, and its conservation within a species may indicate a unique role in the functioning of the system. This sequence does not belong to any known mobile elements and, thus, may serve as a hallmark of Ppa genomes.

Another important area where qPCR detection of SRP is needed is the establishment of the threshold bacterial population necessary for the development of disease symptoms. While the occurrence of SRP-related blackleg, wilting and aerial rot of vegetating potato depends on numerous environmental factors (reviewed in [39]), the development of soft rot in stored ware and seed potato is a consequence of a latent infection of the tuber surface. The incidence of soft rot, as a minimum, correlates with the population of SRP as revealed by laboratory testing. Most in vitro experiments described in the literature use an application of 10^6^–10^7^ cfu/mL aliquots of SRP suspensions applied to unprotected potato tissue (tuber slices) to establish the stable development of soft rot symptoms. This work reports that, starting from almost negligible values, the population of Ppa grew fast at room temperature and reached ~10^6^ cfu/mL, resulting in tissue rotting in a few days. On the other hand, undamaged potato tubers with a latent SRP population 10^4–^10^6^ cfu/mL on the skin revealed no signs of soft rot being stored in proper warehouse conditions (4–7 °C) [40]. Therefore, the monitoring of the bacterial insemination of the tubers may help to estimate the risk of soft rot development in the stored tubers and to reveal the dangerous threshold for each particular SRP species. The designed assay has been shown to be sensitive enough to detect Ppa within the range of natural latent infection level (10^2^–10^5^ cfu). Thus, this analysis is suitable for assessing the quality of potatoes and diagnosing the likely development of rot.

The reported protocol, based on the genomic analysis of an ample amount of recent GenBank data, was successfully tested and demonstrated high sensitivity and suitability for in vivo testing. The species-specific sequence revealed is not only unique to *Pectobacterium parmentieri,* but is also a part of a functional gene which can be important for pathogenic lifestyle of this economically important plant pathogen. The high specificity of the developed assay is particularly important for efficient phage application in the biocontrol of plant diseases caused by SRP bacteria.

## 4. Materials and Methods

### 4.1. Phylogenetic Analysis

Bacterial genomes were downloaded from the NCBI GenBank bacterial database (ftp://ftp.ncbi.nlm.nih.gov/genbank (accessed on 27 March 2021)). A phylogenetic tree was generated using an UBCG pipeline, based on 92 core genes including 43 ribosomal proteins, nine genes of aminoacyl-tRNA synthetases, DNA processing and translation proteins and other conservative genes. Bootstrap analysis phylogeny was conducted by aligned concatenated sequences of 92 core genes made by UBCG with MAFFT (FFT-NS-x1000, 200 PAM/k = 2). Then, bootstrap trees were constructed using the RAxML program (maximum likelihood method) (GTR Gamma I DNA substitution model). The robustness of the trees was assessed by fast bootstrapping (1000) [41].

Average nucleotide identity (ANI) was computed using orthoANI, with default settings [42].

### 4.2. Search for Species-Specific Sequences and Primer Design

To search for species-specific sequences, custom databases were constructed using BLAST (https://blast.ncbi.nlm.nih.gov/Blast.cgi (accessed on 25 February 2021)). The search for species-specific regions for amplification was carried out using the workflow presented in the previous study [20].

Primers and probes were generated with Primer3Plus (https://primer3.ut.ee/ (accessed on 15 March 2021)) and manually checked for the consistency of melting temperatures and for the absence of hairpins and dimers formation using the functions of Geneious Prime and Primer Biosoft (http://www.premierbiosoft.com/NetPrimer/AnalyzePrimerServlet (accessed on 20 March 2021)).

### 4.3. Bacterial Strains, Media and Culture Conditions

A complete list of bacterial strains engaged in this study, with an indication of their species, year and location of isolation, is shown in Supplementary Table S1. Strains were obtained from the Laboratory of Molecular Bioengineering, IBCh RAS. Pectolytic bacteria were cultivated at 28 °C on 1.5% LB agar. CVP medium was used to assess pectinolytic activity. *E. coli* NovaBlue strain was used for transformation during the preparation of a plasmid. *E. coli* was cultivated at 37 °C on LB agar medium with the addition of ampicillin.

### 4.4. Genomic DNA Isolation

Genomic DNA was isolated using overnight bacterial cultures, using a GeneJET Genomic DNA Purification Kit (ThermoScientific, Waltham, MA, USA), according to the manufacturer’s protocol.

Potato DNA was extracted using a CTAB-based protocol. For this purpose, a piece of peel of 100 mg was mechanically homogenised with a 0.1% sodium pyrophosphate solution. The resulting homogenate was transferred into 1.5 mL tubes and centrifuged. 40 μL of lysozyme solution (100 μL/mL) and 60 μL of 10% SDS solution were added to the sediment, resuspended and incubated at 37 °C for 30 min. Then, 650 µL of 2% STAB was added to the mixture and incubated for another 30 min at 65 °C. Then, the mixture was cooled and 700 μL of chloroform was added, vortexed and precipitated at 12,000 rpm. The supernatant was mixed in a new tube with 600 μL of isopropanol. After subsequent centrifugation, the precipitate was washed twice with 75% ethanol and dried until the volatile solvents completely evaporated, and the resulting DNA was dissolved in water.

The concentration and quality of the extracted DNA was estimated using a NanoProteometer N60 (NanoProteometer, Munich, Germany). After extraction, DNA concentrations were diluted to a single value of 10 ng/μL.

### 4.5. PCR Conditions

The conventional PCR was carried out in a volume of 25 μL containing 5 μL of Evrogen ScreenMix (Evrogen, Moscow, Russia,), 0.35 μM of forward and reverse primers and 60 ng of template DNA. Amplification was performed using a T100 Thermal Cycler (Bio-Rad, Hercules, CA, USA) and in the following conditions: 94 °C for 300 s, then 45 cycles of 94 °C for 10 s, 62 °C for 10 s and 72 °C for 10 s. The resulting PCR products were separated by electrophoresis in 1.5% agarose/TA buffer gel and visualised by ethidium bromide staining. The size of the bands was eluted using a 1 kb DNA Ladder marker (Evrogen).

### 4.6. Plasmid Construction for Sensitivity Assay

For a precise evaluation of PCR sensitivity, we constructed a plasmid containing an insert of the target sequence amplified from the Ppa F149 strain. For this purpose, the product of PCR amplification was purified using ISOLATE II PCR and Gel Kit (Bioline, St. Petersburg, Russia) and cloned to pAL2-T vector using a QuickTA kit (Evrogen). Plasmid DNA used as standard was purified with a QIAprep Spin Miniprep Kit (Qiagen, Hilden, Germany), according to the manufacturer’s instructions. Sanger sequencing of the corresponding region in the resulting plasmid confirmed the correctness of the insert.

### 4.7. qPCR

The qPCR was carried out in a LightCycler 96 (Roche, Basel, Switzerland). Each 35 μL reaction contained 200 μM of each dNTP, 0.2 μM of probe, 0.35 μM of forward and reverse primers and 60 ng of template DNA. The optimised amplification conditions were as listed in Section 4.5. Each reaction was carried out in four replicates. Water was used as a negative control. Plasmid-based internal control was used to exclude false-negative results, as described earlier [43].

The processing of the amplification curves obtained and the calculation of the threshold cycles were carried out using software supplied by Roche. A sensitivity analysis was carried out on serial three ten-fold dilutions of the test plasmid and genomic DNA of strain F149. The resulting samples were analysed by qPCR. For each defined threshold cycle, the mean and standard deviation were calculated using Roche software. To construct the standard curve, the threshold cycles’ mean values were plotted against the concentration of copies of the target sequence in each reaction.

For all values, the standard deviation was calculated.

### 4.8. Testing the Detection System on Artificially Infected Tubers

For the experiment, potato tubers of the most widespread variety, “Gala”, were obtained from a market. They were washed and soaked in a bacterial suspension to infect the tubers, following the same protocol as in a previous study [20]. Then, the tubers were incubated at 28 °C. On days three, four, five and six, DNA was extracted from 100 mg of the infected tuber’s peel, as described in Section 4.4, and analysed by qPCR.

## Figures and Tables

**Table 1:** Primers for amplification of a species-specific region and *P. parmentieri* and the amplicon of ankyrin repeat domain-containing protein.

Type	Sequence
F primer	TAT CGC TGG CTC AGG CAA TT
R primer	TAC GCT GCG CAT ACT TGG AA
Probe	(6-FAM)-CGCCCGGG-(dT-BHQ-1)-GCCCAAGATATGACTT-(Pi)
Amplicon	TATCGCTGGCTCAGGCAATTGAAAAAAACGATGAATCAGAAGTCAAAAAACTTTCAGCTCATACGGACCTAAATCGCCCGGGTGCCCAAGATATGACTTTACTATTCTTCGCGATGCAAAATAGTTATGACAAACAAGCCAAACATTTGTCGATAGTCTCATATTTGGTTAGTGCCGGAGCAAGTCCATTACAGAAAGTTCCAAGTATGCGCAGCGTA

**Table 2:** Mean Cq values for qPCR carried out on serial dilutions of genomic DNA of the *P. parmentieri* F149 and corresponding plasmid. SD is standard deviation.

№	Plasmid			DNA		
	Copies Per Reaction	Cq	SD	Copies Per Reaction	Cq	SD
1	1.4 × 10^10^	8.01	0.12	1.68 × 10^7^	16.29	0.37
2	1.48 × 10^9^	11.83	0.21	1.68 × 10^6^	20.48	0.36
3	1.48 × 10^8^	15.27	0.15	1.68 × 10^5^	24.62	0.01
4	1.48 × 10^7^	19.04	0.71	1.68 × 10^4^	27.20	2.90
5	1.48 × 10^6^	22.90	0.57	1.68 × 10^3^	29.92	1.25
6	1.48 × 10^5^	25.67	1.72	168	34.60	1.80
7	1.48 × 10^4^	29.37	3.07	16.8	36.20	0.37
8	1.48 × 10^3^	32.59	0.70	1.68	-	-
9	148	33.95	1.72	0.16	-	-

**Table 3:** Results of qPCR carried out on material obtained from artificially infected potatoes. APC permease gene of Ppa was detected using developed primers. SD is standard deviation.

Incubation, h	Cq	SD	Copies/mL
Control (120 h)	38.32	0.00	6.92
72	31.03	0.67	1.06 × 10^3^
96	27.38	0.67	1.3 × 10^4^
120	20.76	1.05	1.3 × 10^6^

## Supplementary Materials

The following are available online at https://www.mdpi.com/article/10.3390/plants10091880/s1, Table S1 Selectivity of the designed qPCR method.
