# Supplementary material for: Quantitative Real-Time PCR Assay for the Detection of Pectobacterium parmentieri, a Causal Agent of Potato Soft Rot
Source: Plants (Basel). 2021 Sep 10;10(9):1880. doi: 10.3390/plants10091880 (PMC8468878; doi:10.3390/plants10091880)
Supplement: Supplementary file 1 [file plants-10-01880-s001.zip › plants-1380512-supplementary/Supplementary Table 1 Ppar.pdf]

Supplementary Table S1. Selectivity of the designed qPCR method

PCR methods used to verify the taxonomy of the isolates were the following: *Pectobacterium carortovorum* subsp. *carotovorum* (Pca) - [1], *Pectobacterium atrosepticum* (Pat) - [2], *Pectobacterium brasiliense* (Pbr) - [3], *Pectobacterium parmentieri* (former *P.wasabiae*) - [4], *Dickeya solani* (Dso) –[5], *Dickeya dianthicola* (Ddi) - [6]. These methods do not discriminate between *P.carotovorum* (Pca) and *P. versatile* (Pve) [7], and between *P .wasabiae* (Pwa) and *P. parmentieri* (Ppa). Therefore, the isolates characterized by PCR only are regarded as double designation.

№	Strain designation	Alternative strain designation	Geographical region	Species	Genus/species determination	Assay	Cq
1	F127	B5	Kaluga	Ppa	16S, PCR, MLST	positive	20.01
2	F148	PB20	Moscow	Ppa	NZ_PDDJ00000000.1	positive	21.34
3	F149	PB21	Moscow	Ppa	16S, PCR	positive	19.81
4	F174	536 / I3	Moscow	Ppa	PCR	Positive	22.31
5	F177			Ppa		Positive	20.99
6	F034	B9	Kaluga	Ppa	16S, PCR	positive	18.10
7	F035	B7	Kaluga	Pwa/Ppa	PCR	positive	21.56
8	F178			Ppa/Pwa		negative	-
-9	F007	2892	VKPM	Pwa	16S, PCR, MLST	negative	-
10	F164	PB31	Moscow	Paq	NZ_PJJA00000000.1	negative	-
11	F004	PB72	Moscow	Pat	NZ_PDDK00000000.1	negative	-
12	F041		Moscow	Pat	16S, PCR, MLST	negative	-
13	F048	14	Moscow	Pat	16S, PCR, MLST	negative	-
14	F162	SCRI1043	Scotland	Pat	NC_004547	negative	-
15	F163	21A	Belarus	Pat	NZ_CP009125	negative	-
16	F126		Samara	Pbr	NZ_RRYQ00000000.1	negative	-
17	F128		Samara	Pbr	16S, PCR	negative	-

18	F152	PB29	Moscow	Pbr		negative	-
19	F157	PB38	Moscow	Pbr	NZ_PJDL00000000.1	negative	-
20	F053	B6	Unknown	Pca	16S, PCR	negative	-
21	F118	522 / G2	Moscow	Pca	16S, PCR	negative	-
22	F160	NCPPB312	Denmark	Pca	NZ_JQHJ00000000.1	negative	-
23	F057			Pca		negative	-
24	F110			Pca		negative	-
25	F015	D6	Moscow	Pca/Pve	PCR	negative	-
26	F017	246 / I7	Moscow	Pca/Pve	PCR	negative	-
27	F022	2.4 / F1	Tomsk	Pca/Pve	PCR	negative	-
28	F024	A7	Lithuania	Pca/Pve	PCR	negative	-
29	F025	B9	Moscow	Pca/Pve	PCR	negative	-
30	F027	D1	Moscow	Pca/Pve	PCR	negative	-
31	F047	10b	Moscow	Pca/Pve	PCR	negative	-
32	F050	10a	Moscow	Pca/Pve	PCR	negative	-
33	F061	35 / F8	Ethiopia	Pca/Pve	PCR	negative	-
34	F062	246 / I8	Moscow	Pca/Pve	PCR	negative	-
35	F063	D3	Unknown	Pca/Pve	PCR	negative	-
36	F064	520 / F6	Moscow	Pca/Pve	PCR	negative	-
37	F073	5	Unknown	Pca/Pve	PCR	negative	-
38	F077	B6	Ethiopia	Pca/Pve	PCR	negative	-
39	F092	E2	Moscow	Pca/Pve	PCR	negative	-
40	F099	518 / E8	Moscow	Pca/Pve	PCR	negative	-

41	F111	301 / I8	Moscow	Pca/Pve	PCR	negative	-
42	F121	C8	Moscow	Pca/Pve	PCR	negative	-
43	F124	2019.14	Tver	Pca/Pve	PCR	negative	-
44	F133	C1	Tyumen	Pca/Pve	PCR	negative	-
45	F136		Bryansk	Pca/Pve	PCR	negative	-
46	F051			Pca/Pve	PCR	negative	-
47	F067			Pca/Pve	PCR	negative	-
48	F108			Pca/Pve	PCR	negative	-
49	F002	PB69	Moscow	Pve	NZ_PDVY00000000.1	negative	-
50	F003	PB70	Moscow	Pve	NZ_PDVZ00000000.1	negative	-
51	F016		Ryazan	Pve	NZ_RRYR00000000.1	negative	-
52	F018		Moscow	Pve	NZ_PDVV00000000.1	negative	-
53	F020	523 / I6	Moscow	Pve	16S, PCR	negative	-
54	F021	526 / I4	Moscow	Pve	16S, PCR	negative	-
55	F040	524 / H4	Moscow	Pve	16S, PCR	negative	-
56	F131	12a	VIZR	Pve	NZ_PDVW00000000.1	negative	-
57	F135	2.1 / F5	Moscow	Pve	NZ_PDVX00000000.1	negative	-
58	F109	35 / E6	Ethiopia	Ppo	NZ_RRYS00000000.1	negative	-
59	F171	2019.19-4	Moscow	Ppo	16S, MLST	negative	-
60	F014	B1	Tula	Ddi	PCR	negative	-
61	F056	A4	Moscow	Ddi	PCR	negative	-
62	F071	C3	Moscow	Ddi	PCR	?	-
63	F072	B6	Moscow	Ddi	PCR	negative	-

64	F090	18b	Kaluga	Ddi	16S, MLST	negative	-
65	F097	1913 / H9	Moscow	Ddi	PCR	negative	-
66	F117	C9	Bryansk	Ddi	PCR	negative	-
67	F119	F8	Moscow	Ddi	PCR	negative	-
68	F120	C7	Samara	Ddi	PCR	negative	-
69	F032			Ddi		negative	-
70	F101			Ddi	PCR	negative	-
71	F116			Ddi		negative	-
72	F012	Dfil	Voronezh	Dso	NZ_PGOJ00000.1	negative	-
73	F043			Dso	PCR	negative	-
74	F102			Dso	PCR	negative	-
75	F155			Dso	PCR	negative	-
76	F082	H4	Moscow	Dickeya sp.	16S, PCR	negative	-
77	F096	F9	Samara	Dickeya sp.	16S, PCR	negative	-
78	F019	I9	Moscow	Advenella sp.	16S	negative	-
79	F023	A1	Moscow	Advenella sp.	16S	negative	-
80	F113		Unknown	Agrobacterium tumefaciens	16S	negative	-
81	F009	2220	Moscow	Clavibacter michiganensis	16S	negative	-
82	F010	2222	Unknown	Clavibacter michiganensis	16S	negative	-
83	F091		Lithuania	Curtobacterium flaccumfaciens	16S	negative	-

84	F094		Ehtiopia	Curtobacterium sp.	16S	negative	-
85	F033	245/G8	Moscow	Morganella sp.	16S	negative	-
86	F074		Unknown	Morganella sp.	16S	negative	-
87	F039	A8	Lithuania	Pseudomonas sp.	16S	negative	-
88	F052		Moscow	Pseudomonas sp.	16S	negative	-
89	F054	E4	Moscow	Pseudomonas sp.	16S	negative	-
90	F080	C7	Unknown	Pseudomonas sp.	16S	negative	-
91	F093		Lithuania	Pseudomonas sp.	16S	negative	-
92	F114		Unknown	Pseudomonas sp.	16S	negative	-
93	F115	1861a	Belgorod	Pseudomonas sp.	16S	negative	-
94	F123		Moscow	Pseudomonas sp.	16S	negative	-
95	F125		Unknown	Pseudomonas sp.	16S	negative	-
96	F065	442 / H8	Moscow	Serratia sp.	16S	negative	-
97	F059	2354	Unknown	Stenotrophomonas maltophilia	16S	negative	-
98	F036		Unknown	Stenotrophomonas sp.	16S	negative	-
99	F037	20/I3	Moscow	Stenotrophomonas sp.	16S	negative	-
100	F038	1808v	Novgorod	Stenotrophomonas sp.	16S	negative	-
101	F045	A3	Krasnodar	Stenotrophomonas sp.	16S	negative	-
102	F055		Moscow	Stenotrophomonas sp.	16S	negative	-
103	F060		Ethiopia	Stenotrophomonas sp.	16S	negative	-

104	F066		Samara	Stenotrophomonas sp.	16S	negative	-
105	F079	1860b	Voronezh	Stenotrophomonas sp.	16S	negative	-
106	F013	DD	Moscow	Xanthomonas sp.	16S	negative	-
107	F028		Moscow	Xanthomonas sp.	16S	negative	-
108	F030		Moscow	Xanthomonas sp.	16S	negative	-
109	F076	C3	Moscow	Xanthomonas sp.	16S	negative	-

References

1. Kang, H. W.; Kwon, S. W.; Go, S. J. PCR-based specific and sensitive detection of Pectobacterium carotovorum ssp. carotovorum by primers generated from a URP-PCR fingerprinting-derived polymorphic band. *Plant Pathology* **2003**, *52*, 127–133, doi:10.1046/j.1365-3059.2003.00822.x.
2. De Boer, S. H.; Ward, L. J. PCR detection of Erwinia carotovora subsp atroseptica associated with potato tissue. *Phytopathology* **1995**, *85*, 854–858, doi:10.1094/Phyto-85-854.
3. Duarte, V.; De Boer, S. H.; Ward, L. J.; De Oliveira, A. M. R. Characterization of atypical Erwinia carotovora strains causing blackleg of potato in Brazil. *Journal of Applied Microbiology* **2004**, *96*, 535–545, doi:10.1111/j.1365-2672.2004.02173.x.
4. De Boer, S. H.; Li, X.; Ward, L. J. Pectobacterium spp. Associated with Bacterial Stem Rot Syndrome of Potato in Canada. *Phytopathology* **2012**, *102*, 937–947, doi:10.1094/PHYTO-04-12-0083-R.
5. van Vaerenbergh, J.; Baeyen, S.; de Vos, P.; Maes, M. Sequence diversity in the Dickeya flic gene: Phylogeny of the Dickeya genus and taqman® PCR for "D. solani", new biovar 3 variant on potato in Europe. *PLoS ONE* **2012**, *7*, doi:10.1371/journal.pone.0035738.
6. Pritchard, L.; Humphris, S.; Saddler, G. S.; Parkinson, N. M.; Bertrand, V.; Elphinstone, J. G.; Toth, I. K. Detection of phytopathogens of the genus Dickeya using a PCR primer prediction pipeline for draft bacterial genome sequences. *Plant Pathology* **2013**, *62*, 587–596, doi:10.1111/j.1365-3059.2012.02678.x.
7. Portier, P.; Pédron, J.; Taghouti, G.; Fischer-Le Saux, M.; Caullireau, E.; Bertrand, C.; Laurent, A.; Chawki, K.; Oulgazi, S.; Moumni, M.; Andrivon, D.; Dutrieux, C.; Faure, D.; Hélias, V.; Barny, M.-A. Elevation of Pectobacterium carotovorum subsp. odoriferum to species level as Pectobacterium odoriferum sp. nov., proposal of Pectobacterium brasiliense sp. nov. and Pectobacterium actinidiae sp. nov., emended description of Pectobacterium carotovorum and description of Pectobacterium versatile sp. nov., isolated from streams and symptoms on diverse plants. *International journal of systematic and evolutionary microbiology* **2019**, doi:10.1099/ijsem.0.003611.
